# Real Time Monitoring of Engagement with a Text Message Intervention to Reduce Binge Drinking Among Men Living in Socially Disadvantaged Areas of Scotland

**DOI:** 10.1007/s12529-017-9666-z

**Published:** 2017-07-12

**Authors:** Linda Irvine, Ambrose J. Melson, Brian Williams, Falko F. Sniehotta, Andrew McKenzie, Claire Jones, Iain K. Crombie

**Affiliations:** 1 Division of Population Health Sciences, University of Dundee, Dundee, UK; 2 Institute of Health and Wellbeing, University of Glasgow, Glasgow, UK; 3 School of Health & Social Care, Edinburgh Napier University, Edinburgh, UK; 4 Institute of Health and Society, Newcastle University, Newcastle, UK; 5 Health Informatics Centre, University of Dundee, Dundee, UK

**Keywords:** Text messages, Digital behaviour change intervention, Engagement, Disclosure, Reciprocity, Binge drinking, Mobile phone

## Abstract

**Purpose:**

This study identified the extent and nature of engagement with a theoretically based behaviour change text message intervention intended to reduce binge drinking. The data were from a randomised controlled trial tackling binge drinking among socially disadvantaged men.

**Method:**

An intervention, comprising 112 text messages, and based on the principles of the Health Action Process Approach, was delivered to 411 socially disadvantaged men. Participants sent almost 7500 responses to the text messages. Engagement was assessed by whether text message replies showed the intended response to key components of the behaviour change strategy.

**Results:**

The median number of responses to the text messages was 17 per man (range 0–81). Men often gave detailed sensitive personal information about their drinking and the harms it caused them. They also described their attempts at drinking less, the setbacks encountered and the benefits they enjoy when they are successful at cutting down. Specific examples of engagement with the targeted messages include the following: of the 248 men who responded to the prompt on outcome expectancies, most (230) identified potential benefits of cutting down; for intention to reduce drinking, 260 men responded of whom 44% said they had thought about changing; of the 172 men who responded to the question on goal setting, 158 reported personal goals.

**Conclusions:**

The responses showed that most men engaged as intended with the key components of the intervention. Text message interventions should include questions addressing key components of the behaviour change strategy to determine whether there is effective engagement with intervention components.

## Introduction

Text messaging provides a method for delivering behaviour change interventions with the potential to reach large numbers of individuals at low cost. Recent systematic reviews conclude that text messages can promote smoking cessation [1–3], increase adherence to antiretroviral therapy [4, 5] and address weight loss and physical activity [6]. A recent systematic review of text message interventions to reduce alcohol consumption reported that the evidence is promising but preliminary, and recommends further research [7].

Text messaging has become the most common method of electronic communication because it links simplicity with interactivity [8]. More than 90% of people under 65 years in the UK regularly use a mobile phone [9], and users frequently check their phones for messages [10]. Text messages are usually read soon after delivery [11] so individuals receiving an intervention by mobile phone are likely to open and read the messages. Little effort is required by recipients to receive the intervention, and text messages can be accessed at any time. Messages can be read quickly and re-read if desired. People who may not want to commit time for attending appointments or reading leaflets may be willing to receive concise text messages.

Despite a proliferation of text message intervention studies in recent years, there is insufficient evidence to describe the theoretical constructs and mechanisms that lead to modified intentions and subsequent behaviour change. Recent systematic reviews report that few studies provide details on the health behaviour theories that underpin the interventions [12–14], and even among those that give the theoretical basis, few evaluate the effects of the components on behaviour [1, 6, 12, 14].

This study explored the participants’ real time responses to an empirically and theoretically based text message intervention. The aim was to assess the extent of engagement with the intervention. A key element of engagement with digital interventions is intended usage, which has been described as ‘the extent to which individuals should experience the content (of the intervention) to derive maximum benefit from the intervention, as defined or implied by its creators’ [15]. Yardley et al. [16] extend the approach to encompass ‘the process of achieving positive cognitive, emotional, behavioral, and psychologic change’. In this, they emphasise effective engagement which is sufficient to achieve the intended outcome.

This paper builds on the work of our feasibility study which showed that participants often responded to text messages [17, 18]. It uses the data from our recently completed randomised controlled trial [19] to address two research questions:

What was the frequency of responding to the text messages?

Do the responses enable an assessment of effective engagement with the intervention components?

## Methods

This study used data from a four-centre, pragmatic, individually randomised controlled trial of a text message intervention to reduce binge drinking among young to middle-aged socially disadvantaged men [19, 20]. Participants were men aged 25–44 years who were recruited from areas of high deprivation in Scotland. Men were included in the study if they had two or more episodes of binge drinking (>8 UK units in a single session, corresponding to >64 g of alcohol) in the preceding 28 days. This paper focusses on the 411 participants in the intervention group only i.e. those who received the novel intervention. The results of the randomised controlled trial are described elsewhere [19].

### Intervention Design

The alcohol intervention was systematically developed through formative research and piloting with the target population [21]. It was delivered in a series of 112 interactive text messages over 12 weeks. The intervention was based on the principles of the Health Action Process Approach (HAPA) [22], but drew on other theories [23, 24] and incorporated evidence-based behaviour change techniques identified in a synthesis of alcohol intervention studies [25]. It also incorporated components of alcohol brief interventions [26–28] and communication theory [29].

The HAPA provides a useful framework for integrating the effective behaviour change techniques and for guiding decisions on the sequence of delivering these techniques. Thus, the text messages initially guided participants through a motivational phase which focussed on intentions to modify their drinking. This was followed by a volitional phase which encouraged behaviour change using skills such as action planning and coping planning. Self-efficacy was addressed in both phases of the intervention.

### Promoting Interactivity

Two techniques were used to promote interactivity: the style of the text messages and the use of questions. The style of the text messages was informal and conversational, using language tailored to the target group. Some messages included anonymised quotes which were obtained from men in the feasibility study for the trial [21]. These unedited quotes were used to foster engagement through the sharing of experiences of peers. For example, to increase the salience of harms from alcohol, a message read ‘Mark from Edinburgh says “Sometimes I’ve not had enough money left to pay the bills”’.

Twenty-one text messages asked a question. They were designed to reinforce the intervention by encouraging the men to reflect on their drinking and think carefully about changing their behaviour. Questions were designed to assess participants’ engagement with key components of the HAPA model [22]. Five were multiple choice questions which assessed intentions and self-efficacy in changing drinking behaviour. The remaining 16 questions were open ended and designed to encourage reflection and prompt more detailed and personal responses from the participants. The participants did not receive a reply to their responses, nor did they receive a financial incentive or compensation for responding.

### Delivery of the Intervention

The intervention was delivered by an automated computer system, which sent the text messages to participants’ mobile phones in a predetermined sequence. The system was compatible with mobile phones from the most basic to the most recent smartphone. The computer system recorded whether the messages had been delivered to the participants’ phones. The proportion of text messages recorded as delivered to the participants’ mobile phone was monitored as a measure of fidelity of delivery of the intervention. The computer package which delivered the messages also stored electronically text message responses received from participants. All of the responses received were anonymised and collated by the Health Informatics Centre at the University of Dundee.

### Assessing Engagement

The measures of engagement used in this study are derived from the work of Kelders et al. [15] and Yardley et al. [16]. Initially, the extent of usage was quantified by the frequency of text message responses. The responses were then assessed to determine whether the texts were understood. Finally, engagement assessed whether text message replies showed the intended response to key components of the behaviour change strategy.

### Coding of the Responses

A coding system was developed by taking a random sample of 50 responses to every open-ended question. These were independently reviewed and coded by two researchers (AM and LI). The responses were coded as consistent or not consistent with the intended impact of that component of the behaviour change theory. Differences in coding were resolved by discussion with a third member of the team (IKC).

## Results

The 411 men in the intervention group had a mean age of 35 years, just over half were living with a partner and almost one third were unemployed (Table 1). Over 60% of men had high school education only, and over three quarters lived in the most disadvantaged areas of Scotland, as measured by the Scottish Index of Multiple Deprivation (a tool that incorporates several different aspects of deprivation to provide a score based on postcode) [30]. The participants had at least one occasion of binge drinking per week, and mean alcohol consumption was more than 33 UK units per week.

**Table 1:** Characteristics of participants who received the intervention

Factor	*N* = 411 *n* (%)
Age	
25–34 years	221 (53.8)
35–44 years	190 (46.2)
Marital status^a^	
Married/lives with a partner	224 (54.6)
Single	186 (45.4)
Employment status	
Employed	276 (67.2)
Unemployed	135 (32.8)
Highest educational attainment	
High school	250 (60.8)
Vocational qualification/further training	132 (32.1)
University degree	29 (7.1)
Scottish Index of Multiple Deprivation decile	
1–2 (most disadvantaged)	314 (76.4)
≥3	97 (23.6)
Mean number of binge drinking sessions (>8 units on one occasion) in previous 28 days (SD)	6.51 (5.2)
Mean alcohol consumption in previous 28 days (units (SD))	133.0 (132.7)

^a^Marital status not recorded for one man

### Delivery of the Text Messages

A total of 46,032 messages were sent to the 411 participants during the intervention period, of which 95.5% were delivered to their mobile phones. The majority of the men (275) received all of the messages. Of those who missed messages, the median number of undelivered messages per participant was 6 (range 1–112).

### Responses to the Text Messages

**Fig. 1:**
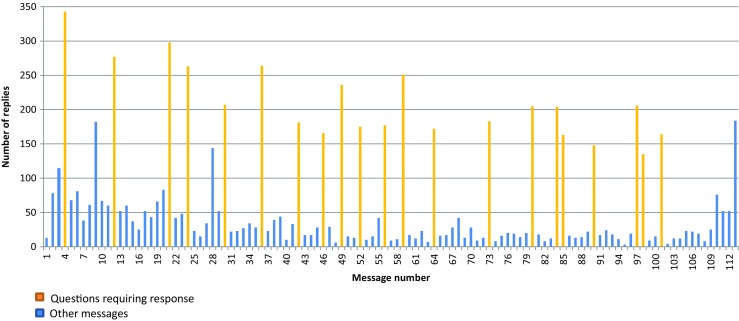
Number of responses to the text message intervention

A total of 7481 responses were received to the 112 messages (Fig. 1). Responses were received from 92% of participants (380 men). The median number of responses per participant was 17 (range 0–81). Although 35% of the participants (142 men) responded more than 20 times, another 33% responded 10 or fewer times. The majority of responses (4416 messages) were to the 21 questions, leaving 3065 (41%) to messages that did not prompt a response. The final column in Fig. 1 represents the 184 responses that were not related to any particular message. These included general comments about the study or how the participants were feeling and also details on change of addresses or phone numbers.

### Motivational and Volitional Messages That Prompted a Response

The nature of the responses provided insight into the ways in which the participants interpreted and reacted to components of the intervention. Almost all of the responses showed positive engagement with the individual text messages. A few responses indicated that specific text messages were not relevant to some men but none were misunderstood. Some men sent more than one response to a prompting text, usually to justify, clarify or extend their initial reply. In the following sections, details are given of the number of responses received, the number of men who replied and the number of men who responded as intended, together with selected verbatim responses.

### Outcome Expectancies

The question ‘Can you think of any reasons why it may be a good idea for you to cut down a bit on your drinking? Please text me your answer’, was asked at the beginning of the third week and was designed to encourage re-evaluation of current drinking behaviour. Posing this as a question encouraged the participants to reflect on any risks and negative outcomes of their drinking and generate reasons for changing which were then committed to text. In total, 263 responses were received from 248 men (60% of the 411 men who were sent the text messages). Most of those who responded (230 men) identified a range of potential benefits, which covered four categories:

- Immediate benefits
  - ‘Be healthier and no more satardays with a sorehead’
  - ‘To remember ur night reduce cost and hangover’.
- Health
  - ‘To be in a better mental and physical shape’
  - ‘my liver function test came bck very high,and the drink is obviously a major reason’.
- Family
  - ‘To try for a baby again with the missus’
  - ‘I want to cut down so I don’t become to ill with the alcohol and want to c my wee boy grow up big and healthy’
- Financial
  - ‘Money, it can be expensive’,
  - ‘Bank balance, productivity on sundays’.

Many men listed several reasons for cutting down:

- ‘A few good reasons for cutting down on drinking for me is better mental and physical health and the ability to enjoy my kids more as a heavy drinking session drains your body’
- ‘My answer for cutting down drinking,save money,better health,u wont get a beer belly,better relationships.<name>’

A few men (*n* = 18) felt that they did not need to cut down:

- ‘I like when I drink. I dont drink to much :-)’
- ‘No I don’t think I need to cut down’.

### Perception of Harm

To increase the salience and personal relevance of harm caused by excessive drinking, men were asked ‘Have you or your mates had any problems caused by alcohol? Please let me know. We’ve all been there’. Almost half (195 men) replied to this question, giving 207 responses in total. A few (*n* = 31) said they had never experienced alcohol-related harm, but 119 men reported personal problems and another 39 only reported the experiences of friends and family members. Some men gave very personal responses describing problems that had occurred from their own drinking and how they had been affected by other people’s drinking:

- ‘Iv spent more than my fair share of nites in police custody, lost the love of my life and a few friends too and been in alot of scraps n all because the drink and boy do i regret it n hope i will learn one day’
- ‘Yeah my step dad was an boozer and would slap me and my mum around. <name>’

Others reported problems that their friends had experienced. These included:

- ‘I’ve had a friend that’s died because of drink. Fell off a balcony in a block of flats’.
- ‘My mate lost everything his family and business all because he decided to drive after a few, he killed a woman and seriously injured her husband in a crash’

### Intention to Change

Intention to change was assessed by a multiple choice question at the end of the third week which asked ‘In the past week have you thought about cutting back a bit on your drinking? Text me (a) yes (b) no (c) maybe’. In total, 264 responses were received from 260 men. Some 44% of those who responded said that they had thought about cutting down, 21% said maybe while 35% of respondents said that they had not thought about it.

### Subjective Norm

To encourage participants to identify people who would approve of their decision to reduce drinking, and thereby increase their motivation to change, a text asked. ‘Can you think of someone who’d be happy if you made a change? What would you hear them say? Please text me your answer’. This question elicited 181 responses from 176 participants. In total, 135 men gave deeply personal responses, identifying parents, partners, family members and friends as people who would be pleased to see a reduction in drinking:

- ‘My kids they would say well done dad’
- ‘My girlfriend would be happy less chance i’d be ending up dead like her father’.
- ‘My gran.youll lose that horrible beer belly’.
- ‘My wife, she’d get more money for shoes!!’

A few men (*n* = 41) could not think of anyone who would be pleased:

- ‘I’ve racked my brain for this question and i can’t think of anyone. Sorry’.
- ‘I dont have anyone that would say i drink too much and need to cut back...’

### Goal Setting and Action Planning

Setting goals and making action plans are important components of the volitional phase of HAPA. Goal setting was introduced in week 5, and participants were asked: ‘If you made a goal to cut down a bit on your drinking, what would it be? Text me your answer’. The 177 responses were received from 172 men, only 14 of whom did not suggest a goal. The 158 men who identified goals to reduce consumption proposed reducing the frequency of drinking occasions or reducing the amount consumed during drinking occasions. The men presented a variety of approaches to drinking less, each one tailored to the individual’s lifestyle:

- ‘Stop Drinking during the week!’
- ‘To just have a tin of juice back at the pub after football on a Saturday instead of a pint which leads onto more pints’
- ‘Stop earlier in the evening or at very least slow down compared to others’
- ‘Buy a 12pack fortnightly instead of weekly’
- ‘To cut out drinkin into early hours of mornin. And avoid awful hangovers’

Action self-efficacy was addressed 2 days later in a multiple choice question which asked ‘How confident are you that you could cut back a bit? Text me back please’. The total of 251 responses to this question came from 241 men. Just over half of these men (51%) expressed very high confidence levels, another 27% were fairly confident, 16% were uncertain and 4% said that they lacked confidence.

When prompted to describe action plans, participants also identified approaches geared to their personal drinking habits. The first of a pair of messages explained: ‘When you make a plan it always works better if you make sure you say: WHEN; WHERE; and HOW you will do it’. This was followed by ‘If you made a plan, what would it be? Text me your answer’. One hundred and sixty-eight men responded to this question, giving 172 responses. Only 20 men had no plan. The remaining men presented plans which varied greatly in content. Some men took a cue from the message and gave a very structured plan:

- ‘Plan- WHEN: tonight. WHERE: watching the footie. PLAN: No more than 3 drinks’.
- ‘When: Saturday night. Where: the golf club. How: go home for tea rather than stay all afternoon and evening’.
- ‘WHEN:monday, WHERE:at home, HOW:not having a can of beer with the nfl game’

Others presented less structured, basic plans which could be interpreted as intentions or aspirations:

- ‘Only drink one night this weekend’
- ‘Im going to not buy beer for at home after the pub so i dont drink when i get in from pub’

During week 8 of the 12-week intervention, following several text messages which discussed relapse and recovery self-efficacy, a multiple choice question asked: ‘If you had an unplanned binge, how confident are you that you could get back on track next time?’ Fewer men responded to this question on maintenance self-efficacy (*n* = 204) than they did on action self-efficacy (*n* = 241). Just over half of those who responded (56%) had very high confidence levels, and 27% were fairly confident.

### Responses to Messages That Did Not Prompt a Reply

Many participants chose to send responses to text messages that did not seek a reply. These spontaneous comments give further evidence of the extent of engagement with the study. Some responses described personal and intimate issues:

- ‘I need to cut back now my wife has told me if I drink one more time when shes at work and I have the kids then shes off’
- ‘My rent arrears are a constant problem for me. I’m building up to a thirty day abstinence throughout June so should begin to clear them off a bit - assuming I go to work once I quit’.
- ‘Think of the hangover or that feeling of ‘the fear’ the next day! It’s not worth it!’

Some of the text messages in the intervention included humour. This appeared to encourage participants to reciprocate with jokes of their own: For example a text message describing how alcohol can affect hormones and cause male breasts or ‘man boobs’ prompted:

- ‘Haha no worries there got solid pecs lol! But hormones I never knew about?’
- ‘Got a beer belly but no moobs. Yet’

However, some men took the message very seriously:

- ‘I didn’t think about the moob thing or hormones. This is good to know as me and my wife want to start a family’.

The definition of binge drinking (>8 UK units of alcohol on a single drinking occasion) also elicited humorous responses, although again, some men made a serious point:

- ‘4 pints is a binge. Geezo that’s harsh:) what’s 8 pints then a disaster??’
- ‘I’ve probably spilt 8 units on an all day session haha’
- ‘Yeah the official definition certainly puts it into perspective. I’m at a wedding right now (on a Thursday!) and I have had three pints already...’

The final three messages thanked the men for taking part, wished them well and reminded them to report changes to their address or phone number. These messages prompted 180 responses in total, many of which expressed gratitude for being part of the study:

- ‘Happy i done this helped me quite a lot so defo glad i took part another sober weekend for me this week’.
- ‘Had my ups and downs but im getting there now. Enjoyed the experience, thank you’
- ‘Thanks for everything it’s been a blast take care’

## Discussion

This study has developed and tested a real time and real world method of assessing the impact of individual text messages within a digital behaviour change intervention. Almost all of the participants gave responses to the text messages which demonstrated that they were fully understood. The responses also showed that men frequently gained positive cognitive, emotional and behavioural benefit from the text messages, indicating effective engagement with the intervention. The frequency of disclosure of personal details, particularly to those text messages that did not prompt a response, confirms a high level of trust which is required for engagement.

Effective engagement was assessed using the approach developed for digital behaviour change interventions [15, 16]. We have applied this approach to components of the intervention, determining whether the responses indicate that the intended outcome of the component has been achieved. This study has shown that the important distinction between engagement with the intervention components and more general engagement with the study also applies to evaluation of the impact of intervention components. Some questions elicited responses that demonstrated positive cognitive and behavioural benefits, while others indicated no such benefit, but still motivated participants to reply.

An important finding from this study is the high levels of responses from the men. The study took advantage of the social rules for text messaging where the receipt of a message is likely to prompt a response [31]. Socially disadvantaged people are more likely to use text messaging and send and receive a higher number of text messages than people with higher education and income [32]. The intervention was tailored for men which could have encouraged feelings of camaraderie or cohesiveness which are found in single gender studies [33–35].

Another factor which may have encouraged a high level of response is that the messages were constructed to be conversational, using unedited quotes from our feasibility study [18, 21]. These techniques were intended to impart the feeling of being part of a dialogue, using participants’ own language to describe experiences and situations familiar to them. The aim was to make the intervention appear authentic and credible. Dialogue moves away from delivering information in a passive way to learning through interactive engagement [36]. Taking part in a dialogue also allows individuals to learn about others’ values and interests [37]. Petralgia [38] reports that ‘contemporary learning theory suggests that learners’ perceptions of authenticity are critical because learning is embedded in our everyday experience of the world rather than in the world of formal information dissemination’. Authenticity ensures that the recipients understand not only cognitively but also emotionally how the information can relate to their lives.

Interactivity was encouraged by sending messages that prompted a response. Text message questions have been used in previous trials to promote interactivity [39, 40], but this study extended the use of this technique to reinforce key components of the behaviour change strategy and to monitor participants’ engagement in real time [18]. Responses to the questions on the key components of the behaviour change strategy indicated that participants responded to the intervention in the way that was intended.

A further notable finding is that the men frequently disclosed sensitive personal information. Self-disclosure can play a role in the development of relationships [41], and reciprocal disclosure has been shown to lead to more positive interpersonal outcomes, such as greater responsiveness, enjoyment and perceptions of being liked, than non-reciprocal disclosure [37, 42]. Internet surveys have shown that self-disclosure by the experimenter led to greater self-disclosure by participants [43]. The inclusion of anonymised quotes from participants in our feasibility study [21] may have encouraged the participants in this study to disclose personal details of their own.

The use of humour may have encouraged responses to texts and self-disclosure. It appeared to appeal to the participants, who responded with jokes and often self-deprecating humour. These matched the style of messaging in the intervention [43] because although they made jokes, many of the responses also had a serious side. Gray et al. suggest that laughter increases people’s willingness to disclose information about themselves [44].

At every step, the participants appeared to translate the messages to fit with their own lives. Instead of responding in general terms, the men gave specific, personal details. For example, discussion on the potential benefits of reducing drinking elicited responses on how their personal health could be improved or how their children could benefit. Similarly, for action planning, changes to be made were specific to the individuals’ drinking patterns.

This study has limitations. A key issue is the interpretation of non-response to text messages. Although 92% of the participants responded to the messages, some men replied on a few occasions only. This does not necessarily show a lack of engagement with the intervention. There may be many reasons why participants did not respond. Participants may not have had time to compose a response or may not have had credit on their phones. Some men may have reacted as intended but thought it was not necessary to respond. It is likely that the total number of responses underestimates the true level of engagement with the study. The nature of non-responders has had little attention in text message studies, but is well described in online discussion groups, where the term lurkers is use to describe non-responders [45]. Lurkers are a special group of web-site users who regularly log in to online communities but seldom post messages [46]. The literature suggests that 90% of participants do not post messages, 9% do so occasionally and only 1% do so regularly [47]. However, lurkers frequently read the online information and gain benefit from it [46, 48]. This issue needs to be addressed in text message interventions.

A further limitation is the brevity of individual text messages responses. Although some men gave extended explanations, most gave succinct responses. Further, the responses to these questions were often varied. For example, in response to the question on action planning, some men provided detailed action plans and others made statements such as ‘not buying any alcohol with my weekly shop!’ To cope with this brevity and diversity, a simple binary code (responded as: intended or not intended) was used.

This study has limited generalisability. Although these methods were acceptable to young to middle-aged socially disadvantaged men, the study provides no evidence that it would be attractive to other social groups. Nevertheless, the success of the study, in reaching and effectively engaging socially disadvantaged men in a behaviour change intervention, has implications for a range of public health interventions, not binge drinking alone.

## Conclusions

This study has shown that the responses to text message interventions can identify whether the components of a behaviour change intervention achieve their intended outcomes. Responses to the questions on the key components of the HAPA model indicated that participants had understood the health messages that were being delivered and responded to them in the way that was intended. Participants appeared to translate the intervention to fit with their own lives and experiences. They were willing to disclose sensitive personal information indicating that the study had gained their trust. They responded with humour which showed they were at ease with the study processes. Text message interventions should include questions addressing key components of the behaviour change strategy to determine whether there is effective engagement with intervention components.
